# Serial measurement of neuron specific enolase improves prognostication in cardiac arrest patients treated with hypothermia: A prospective study

**DOI:** 10.1186/1757-7241-20-6

**Published:** 2012-01-29

**Authors:** Christian Storm, Jens Nee, Achim Jörres, Christoph Leithner, Dietrich Hasper, Christoph J Ploner

**Affiliations:** 1 Department of Nephrology and Medical Intensive Care Medicine, Charité Universitätsmedizin Berlin, Campus Virchow-Klinikum, Augustenburger Platz 1, 13353 Berlin, Germany; 2 Department of Neurology, Charité Universitätsmedizin Berlin, Campus Virchow-Klinikum, Augustenburger Platz 1, 13353 Berlin, Germany

**Keywords:** NSE, kinetic, prognostication, cardiac arrest, hypothermia

## Abstract

**Background:**

Neuron specific enolase (NSE) has repeatedly been evaluated for neurological prognostication in patients after cardiac arrest. However, it is unclear whether current guidelines for NSE cutoff levels also apply to cardiac arrest patients treated with hypothermia. Thus, we investigated the prognostic significance of absolute NSE levels and NSE kinetics in cardiac arrest patients treated with hypothermia.

**Methods:**

In a prospective study of 35 patients resuscitated from cardiac arrest, NSE was measured daily for four days following admission. Outcome was assessed at ICU discharge using the CPC score. All patients received hypothermia treatment for 24 hours at 33°C with a surface cooling device according to current guidelines.

**Results:**

The cutoff for absolute NSE levels in patients with unfavourable outcome (CPC 3-5) 72 hours after cardiac arrest was 57 μg/l with an area under the curve (AUC) of 0.82 (sensitivity 47%, specificity 100%). The cutoff level for NSE kinetics in patients with unfavourable outcome (CPC 3-5) was an absolute increase of 7.9 μg/l (AUC 0.78, sensitivity 63%, specificity 100%) and a relative increase of 33.1% (AUC 0.803, sensitivity 67%, specificity 100%) at 48 hours compared to admission.

**Conclusion:**

In cardiac arrest patients treated with hypothermia, prognostication of unfavourable outcome by NSE kinetics between admission and 48 hours after resuscitation may be superior to prognostication by absolute NSE levels.

## Background

The recommended examinations proposed by the American Academy of Neurology (AAN) for prognostication in patients after cardiac arrest have mainly been evaluated in the era prior to hypothermia [1]. However, recent studies indicate that mild therapeutic hypothermia modifies the prognostic significance of clinical findings, NSE levels, and electrophysiological testing in patients resuscitated from cardiac arrest [2-6]. This has generated the necessity to re-evaluate all prognostic markers in patients treated with hypothermia. In particular, NSE cutoff levels and their temporal dynamics have only rarely been investigated in these patients so far. In this study, we prospectively investigated serum NSE levels and NSE kinetics in 35 cardiac arrest patients treated with hypothermia.

## Methods

The study protocol was approved by the local ethics committee on human research and was conducted in accordance with the guidelines of the Declaration of Helsinki. Written informed consent to the use of routine clinical data is part of the standard contract between patients and the University Hospital Charité Berlin and was obtained from patients or their legal representatives.

Thirty-five consecutive patients resuscitated from cardiac arrest were included in the study. All patients received mild therapeutic hypothermia (MTH) irrespective of the initial cardiac rhythm. Baseline characteristics of the patients are summarized in Table 1. Therapeutic hypothermia was initiated immediately after admission with an intravenous infusion of cold saline (4°C, 1000-1500 ml bolus) followed by surface cooling with commercially available non-invasive devices (ArcticSun2000^® ^Medivance, USA). The target temperature (33°C) was maintained for 24 hours. Intravenous sedation and analgesia were induced in all patients by a combination of midazolam (0.125 mg/kg/h) and fentanyl (0.002 mg/kg/h) with dose adjustment as needed. In order to prevent shivering, patients received muscle relaxation by repetitive administration of pancuronium (0.1 mg/kg).

**Table 1:** Baseline characteristics given as absolute numbers and percent or median and interquartile range (IQR).

Variable	
Age (years)	63 (51-71)
Female sex	12 (43.3)
APACHE Score	28 (22-33)
*Location of cardiac arrest*	
Out-of-hospital	28 (80)
In-hospital	7 (20)
*Cause of cardiac arrest*	
AMI	16 (45.7)
Primary arrhythmia	10 (28.6)
Respiratory	9 (25.7)
Shockable rhythm	15 (42.9)
Time to ROSC (min)	19 (12-30)
Total epinephrine dose (mg)	2.5 (1.5-5)
Length of ICU stay (days)	7 (4-22)
Time on ventilator (hours)	161 (116-519)
*Neurological outcome*	
CPC 1-2	11 (31.5)
CPC 3-5	24 (68.6)

APACHE: Acute Physiology and Chronic Health Evaluation, AMI: acute myocardial infarction, ROSC: return of spontaneous circulation, ICU: intensive care unit, CPC: cerebral performance category.

NSE serum levels were measured on admission and daily until day four with an enzyme immunoassay (Elecsys 2010, Roche Diagnostics GmbH, Mannheim, Germany). None of the patients included in this study received acute surgical interventions, implantation of IABP or renal replacement therapy within the first three days. In addition all blood samples underwent visual inspection before testing to reduce the potential risk of missing relevant haemolysis and to avoid false elevated results of NSE. Clinical outcome was assessed at the time of discharge from ICU by means of the Pittsburgh cerebral performance category (CPC) [7]. All patients were discharged directly to a neurological rehabilitation facility. As in previous studies of outcome following cardiac arrest, a CPC score of 1-2 was classified as favourable neurological outcome, whereas a CPC score of 3-5 was classified as unfavourable outcome. In detail the categories define normal function (CPC 1) minor disability (CPC 2), severe disability (CPC 3), coma (CPC 4) and death (CPC 5). NSE levels were determined in 35 patients at admission and then every 24 hours. Subsequently, the NSE dynamics between day one and day three (absolute increase and percentage increase) was analysed in 33 patients (data from two patients were incomplete). The first sample was taken directly on admission (direct admission to ICU from EMS), therefore the time between admission and second sample is not exactly 24 hours as it is in all other time increments. NSE levels were not blinded to the physicians in charge but any decisions on therapy withdrawal were never based on biochemical markers alone. Neurological assessment was carried out by an external neurologist and the decision to withhold or withdraw further therapies was taken in all patients considering the results of clinical signs, biochemical and electrophysiological testing.

Since data were not normally distributed, descriptive statistics were done by using medians and inter-quartile ranges (IQR). The software Statistical Package for the Social Sciences (SPSS Inc.; Chicago, Illinois) was used for statistical analysis. Non-parametric test was used for comparison of data. Sensitivities and specificities of different NSE thresholds for prediction of neurological outcome were visualized by receiver operating curves (ROC).

## Results

### NSE levels on admission and at 24, 48 and 72 hours

**Figure 1:**
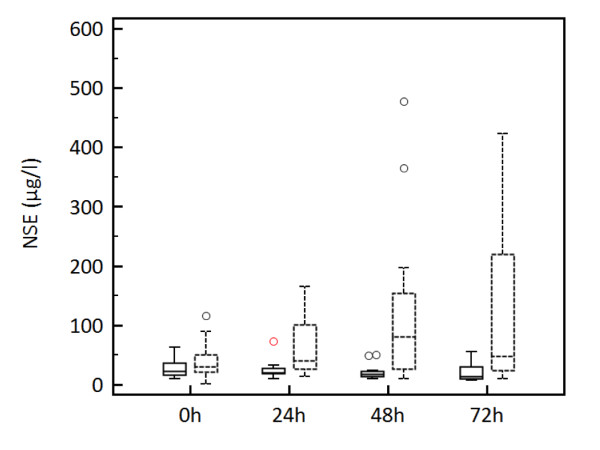
**Boxplots with median and percentiles (25/75) of serum NSE levels for favourable outcome (CPC 1-2, solid boxes) and unfavourable outcome (CPC 3-5, dashed boxes) at different time points (p < 0.05)**.

Baseline characteristics of the study population are given in Table 1. Cutoff values of absolute NSE level and NSE kinetics obtained at different time points after cardiac arrest are summarized in Table 2. For absolute NSE levels, the highest predictive value was found for NSE at 48 hours with a cutoff level of 54.5 μg/l (specificity 100%, sensitivity 58%). At 72 hours, the cutoff level was similar (57.2 μg/l), but sensitivity was markedly lower (47%). Sensitivity was still lower for NSE levels on admission and at 24 hours (8% and 30%, respectively; Table 2). Median NSE levels were significantly lower when adjusted for favourable outcome (CPC 1-2) and unfavourable outcome (CPC 3-5) depending on the different time points of measurement after cardiac arrest (p < 0.05; Figure 1).

**Table 2:** AUC values and cutoff values with 100% specificity for prognostication of unfavourable outcome.

NSE (hours)	cutoff (μg/l)	specificity %	sensitivity %	AUC	95% CI
0	75.5	100	8	0.614	0.41-0.82
24	73.6	100	30	0.804	0.65-0.96
48	54.5	100	58	0.848	0.72-0.98
72	57.2	100	47	0.824	0.67-0.98
absolute difference (increase)					
0/48	7.9	100	63	0.784	0.63-0.93
24/48	25.1	100	39	0.673	0.50-0.84
24/72	28.1	100	37	0.711	0.52-0.90
percentage difference (increase)					
0/48	33.1	100	67	0.803	0.66-0.95
24/48	58.0	100	43	0.671	0.50-0.84
24/72	74.9	100	42	0.760	0.58-0.95

AUC: area under the curve, CI: confidence interval.

### NSE kinetics

**Figure 2:**
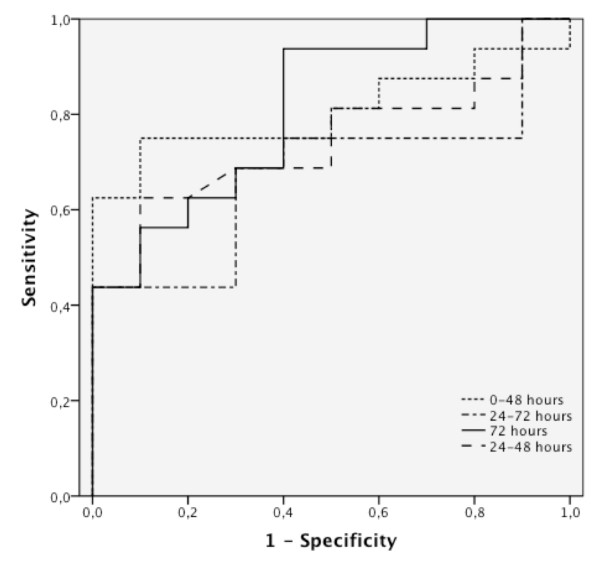
**ROC analysis of NSE serum levels at different time points of measurement (absolute differences)**.

The highest predictive value was found for a relative increase in NSE of 33.1% between admission and at 48 hours (specificity 100%, sensitivity 67%). A similar predictive value was obtained for an absolute increase in NSE between admission and at 48 hours with a cutoff level of 7.9 μg/l (specificity 100%, sensitivity 63%). The predictive value of the difference between NSE levels at 24 and 72 hours was markedly lower (sensitivity 37% for absolute and 42% for relative increase) as well as for the difference between 24 and 48 hours (sensitivity 39% for absolute and 43% for relative increase; Figure 2). Cutoff values and area under the curve (AUC) for NSE level changes (absolute and percentage) are presented in Table 2.

## Discussion

Prognostication of outcome in cardiac arrest survivors treated with hypothermia may be different from non-hypothermia treated patients [2,3,8]. Accordingly, recent data suggest that guideline NSE thresholds for outcome prediction need to be re-evaluated in patients treated with hypothermia. Our results indicate that in these patients, repeated NSE measurements and calculation of NSE kinetics may be superior compared to absolute NSE levels determined on day one, two or three after cardiac arrest, as recommended in the current guidelines of the American Academy of Neurology [1]. Instead, reliable outcome prediction by repeated NSE measurements appears to be possible as early as 48 hours after cardiac arrest.

In a sample of 97 patients, we have previously shown that individual patients may survive with good neurological outcome despite NSE levels of up to 80 μg/l measured 72 h after cardiac arrest. The greatest therapeutic effect of hypothermia was observed in patients with NSE levels between 20 and 80 μg/l, whereas in patients with high (> 80 μg/l) or low (< 20 μg/l) NSE levels the effect was smaller [2]. In a recent study by Daubin and colleagues an even higher cutoff of 97 μg/l was discovered for prediction of unfavourable outcome [5]. In the present study, the sample is smaller and consists of 35 patients, eleven of which had a favourable outcome. Therefore, the cutoff levels obtained must be interpreted with caution and may be higher in individual subjects. In a sub-analysis of the HACA trial, Tiainen and colleagues analyzed the effect of therapeutic hypothermia on serum NSE levels 24, 36 and 48 hours after cardiac arrest [9]. NSE levels were significantly lower in patients treated with hypothermia. After 24 hours, the NSE cutoff for prognostication of unfavourable outcome was 31.2 μg/l (specificity 96%, sensitivity 22%, AUC 0.72). At 48 hours, median NSE level in the hypothermia group was only slightly lower compared to the control group (median 7.9 μg/l and 8.6 μg/l; respectively). The 48 hours NSE cutoff for prognostication of unfavourable outcome was 25.0 μg/l (specificity 96%, sensitivity 25%, AUC 0.80). Current results by Mörtberg et al. conflict with other studies, in that protein S-100 was superior to NSE at all points of measurement (1, 2, 6, 12, 24, 48 and 96 hours) in predicting poor outcome, and peak sensitivity of S-100 was very early after 24 hours (87% sensitivity, 100% specificity) [10]. The authors interpret their novel data with care due to small sample size but argue that compared to other trials all types of rhythms have been included in their analysis.

Compared to these data, our analysis for prognostication of unfavourable outcome revealed a higher sensitivity and a greater AUC especially for the NSE increase from admission to 48 hours. Furthermore, a trend towards a reduced discriminatory power of serum NSE levels for unfavourable neurological outcome under hypothermia treatment was found by Tiainen et al. with an AUC of 0.89 in the control group compared to an AUC of 0.80 in patients treated with hypothermia [9]. This may suggest that the relationship between NSE and neurological outcome is indeed modified by hypothermia treatment.

Many different cutoff levels have been published so far [5,11-15]. The comparability of absolute NSE values for prediction of neurological outcome seems limited as cutoffs are presumably affected by multiple factors including time to NSE measurement, laboratory immunoassay, cause of cardiac arrest, in/out-of-hospital cardiac arrest, time to ROSC and outcome definition. In addition, the ideal time point for measurement of NSE in patients treated with MTH for predicting outcome is still unclear. The major limitation concerning comparability of the different results is a lack of a gold standard in NSE testing (best time; best and standardized laboratory method). This was addressed already by almost all other authors and underlines the need for more data towards prognostication with biomarkers before being incorporated in the guidelines [10,16,17]. Finally the possible influence of the cooling method (device controlled vs. alternative methods), which might lead to a different temperature performance, could possibly influence NSE kinetic as well.

However, our results indicate that a serial measurement might be a better predictor for patients undergoing hypothermia treatment.

Our results suggest dynamic changes in NSE serum levels after resuscitation. While the pathophysiological mechanisms underlying NSE release with global cerebral ischemia following cardiac arrest are not precisely known, experimental studies of focal cerebral ischemia suggest that NSE release may reflect several steps in stroke evolution. A first peak is thought to reflect a rapid NSE release out of the initially damaged tissue, followed by a second peak reflecting secondary mechanisms of brain damage, ongoing neuronal cell death or persistent disturbance of the blood-brain barrier [11,12,18]. Dynamic changes of NSE serum levels in patients after cardiac arrest may similarly reflect distinct but overlapping mechanisms of neuronal damage that may be differentially affected by hypothermia compared to non-hypothermia treatment. It appears therefore possible that the well-characterized kinetics of serum NSE changes in non-hypothermia patients differs quantitatively and qualitatively from the kinetics observed here [1]. This may at least partly explain differences between our observations and previous trials in larger samples of non-hypothermia patients.

Our study has several limitations that need to be addressed. In this pilot study the number of patients is too small to make definite conclusions about reliable outcome prediction with calculated NSE changes. In addition the population studied is heterogeneous concerning location of the occurrence of cardiac arrest and initial rhythm. Results were obtained in a single center and we did not collect data on neurological long-term outcome. We nevertheless deem that this should not put the central findings of our study in question, as it has been shown that CPC scores at ICU discharge undergo only minor changes in the following six months [13,19,20]. All survivors after cardiac arrest were discharged from ICU directly to a neurological rehabilitation facility; therefore the CPC used for analysis of the group included in this study is the CPC at discharge from hospital. Another important problem is the fact that all studies on prognostication after cardiac arrest are susceptible to "self-fulfilling prophecies". Indeed, in our study, NSE levels were not blinded to the physicians in charge. However in our hospital, decisions on therapy withdrawal are never based on biochemical markers alone. Neurological assessment was carried out by an external neurologist and the decision to withhold or withdraw further therapies was taken in all patients considering the results of clinical signs, biochemical and electrophysiological testing. 11 out of 19 patients with CPC 5 died after therapy withdrawal due to suspected infaust prognosis.

## Conclusion

Taken together, the findings in our study suggest that serial measurement of serum NSE may be superior to measurement at a single time point. Determination of NSE kinetics across a temporal window as short as 48 hours appears to provide reliable information on outcome of cardiac arrest patients treated with hypothermia. Larger trials are needed to establish precise prognostic cutoff levels for NSE kinetics. Until these data are available, absolute NSE cutoff levels for prognostication after cardiac arrest should be interpreted with caution.

## List of abbreviations

AAN: American Academy of Neurology; AUC: area under the curve; CPC: cerebral performance category; ICU: intensive care unit; IQR: inter-quartile range; MTH: mild therapeutic hypothermia; NSE: neuron specific enolase; ROC: receiver operating curve; ROSC: return of spontaneous circulation.

## Competing interests

The authors declare that they have no competing interests.

## Authors' contributions

CS made substantial contributions to conception and design of the study, data acquisition, analysis and interpretation of the results. JN, AJ and CL participated in data collection and drafted the manuscript. DH performed statistical analysis and made substantial contributions to the study design and coordination. CP participated in the design and conception, interpretation of the results and helped draft the manuscript. All authors read and approved the final manuscript.
